# Antibacterial and Antibiofilm Potential of Chlorophyllin Against *Streptococcus mutans* In Vitro and In Silico

**DOI:** 10.3390/antibiotics13090899

**Published:** 2024-09-20

**Authors:** Seemrose Khan, Ihtisham Ul Haq, Imran Ali, Abdul Rehman, Mazen Almehmadi, Meshari A. Alsuwat, Tariq Zaman, Muhammad Qasim

**Affiliations:** 1 Department of Microbiology, Kohat University of Science & Technology, Kohat 26000, Pakistan; rose050297@gmail.com (S.K.); abdulrehman@kust.edu.pk (A.R.); 2 Department of Physical Chemistry and Technology of Polymers, Silesian University of Technology, 44-100 Gliwice, Poland; ihaq@polsl.pl; 3 Joint Doctoral School, Silesian University of Technology, 44-100 Gliwice, Poland; 4 Postgraduate Program in Technological Innovation, Federal University of Minas Gerais, Belo Horizonte 31270-901, Brazil; 5 Department of Botany, Kohat University of Science & Technology, Kohat 26000, Pakistan; drimranali@kust.edu.pk; 6 Department of Clinical Laboratory Sciences, College of Applied Medical Sciences, Taif University, P.O. Box 11099 Taif, Saudi Arabia; mazenn@tu.edu.sa (M.A.);; 7 Department of Pediatrics & Human Development, College of Human Medicine, Michigan State University, East Lansing, MI 48824, USA; zamantar@msu.edu

**Keywords:** dental caries, *Streptococcus mutans*, biofilms, chlorophyllin, antibacterial activity, proteins docking, ADMET analysis

## Abstract

Background: *Streptococcus mutans* is a leading causative agent of dental caries and exerts pathogenicity by forming biofilms. Dental caries continues to be a significant public health issue worldwide, affecting an estimated 2.5 billion people, showing a 14.6% increase over the past decade. Herein, the antibacterial potential of Chlorophyllin extracted from *Spinacia oleracea* was evaluated against biofilm-forming *S. mutans* via in vitro and in silico studies. Methodology: The antimicrobial activity of chlorophyllin extract against *S. mutans* isolates was tested using the agar well diffusion method. Chlorophyllin extract was also tested against biofilm-forming isolates of *S. mutans*. Chlorophyllin was docked with the antigen I/II (AgI/II) protein of *S. mutans* to evaluate its antimicrobial mechanism. The chemical structure and canonical SMILES format of Chlorophyllin were obtained from PubChem. Additionally, adsorption, distribution, metabolism, excretion, and toxicity (ADMET) analyses of Chlorophyllin were performed using ADMETlab 2.0 to assess its pharmacokinetic properties. Results: An agar well diffusion assay revealed that all *S. mutans* isolates were susceptible to Chlorophyllin extract and showed a variety of inhibition zones ranging from 32 to 41 mm. Chlorophyllin reduces the biofilm strength of four isolates from strong to moderate and six from strong to weak. The antibiofilm potential of Chlorophyllin was measured by a reduction in the number of functional groups observed in the Fourier Transform Infrared Spectrometer (FTIR) spectra of the extracellular polymeric substance (EPS) samples. Chlorophyllin showed binding with AgI/II proteins of *S. mutans*, which are involved in adherence to the tooth surface and initiating biofilm formation. The ADMET analysis revealed that the safety of Chlorophyllin exhibited favorable pharmacokinetic properties. Conclusions: Chlorophyllin stands out as a promising antibacterial and antibiofilm agent against biofilm-forming *S. mutans*, and its safety profile highlights its potential suitability for further investigation as a therapeutic agent.

## 1. Introduction

Many bacterial pathogens including *Streptococcus mutans* are harbored in the human oral cavity and are associated with different infectious complications such as dental caries (DC) [1]. One-third of the global population is affected by DC, which leads to direct treatment costs amounting to approximately USD 300 billion for the worldwide economy on an annual basis [2,3]. In the drug resistance area, DC emerges as one of the most highly complicated problems in the world [4]. Although the prevalence of DC is reported to have decreased among 12-year-old children in developed countries, the worldwide occurrence of untreated DC remains persistently high [5]. Saliva exchange, mother-to-child transmission, and contaminated surfaces are the most common routes of *S. mutans* transmission [6]. The cariogenic properties allow *S. mutans* to colonize the oral cavity under acidic conditions [7]. *S. mutans*’ adherence on the tooth surface is facilitated by acid and water-insoluble glucan in the presence of sucrose [4] using surface proteins such as antigen I/II as adherence factors. *S. mutans* uses the AgI/II protein in its attachment to the tooth surface and biofilm formation using salivary glycoproteins as binding proteins [8]. It has been reported that the biofilm-forming ability of Ag I/II-deficient mutants is significantly low compared to wild-type *S. mutans* [9]. Ag I/II structure is uniformly preserved across all variants in this protein family; this complex, multidomain protein consists of 1500–1566 amino acid residues and is considered a vital target protein for antimicrobials [9].

The biofilm-forming ability increases caries’ persistence, resulting in high antimicrobial resistance and deficient immune responses [10]. Biofilm is a complex mixture of extracellular polysaccharides (EPS), and the microbial community formed on the tooth surface plays an essential role in DC. The EPS matrix in biofilm is more resistant to conventional antibiotics and host defenses than planktonic cells. These *S. mutans* biofilms, in the presence of sucrose medium, produce lactic acid in large quantities as the end product of the bacterial metabolic process. The lactic acid produced by bacteria lowers the pH and dissolves the hydroxyapatite crystals in the dental enamel, ultimately leading to cavities [11]. Antibiotics are often prescribed to treat dental caries to prevent recurrent dental infections. However, frequent exposure to commonly available antibiotics has led to resistance, making bacterial dental infections challenging to treat [12]. It has been observed that antimicrobial resistance in streptococci has been significantly increased, leading to the failure of antimicrobial therapy [6]. DC associated with cariogenic *S. mutans* can develop resistance against antimicrobial agents, including antimicrobial peptides [13].

With antibiotic resistance being a global concern, scientists now opt for natural compounds as safe alternative therapeutic strategies against bacterial biofilms [14]. Recently, there has been a growing interest in identifying alternative antimicrobial compounds, including plant-derived products such as Chlorophyllin, to combat antibiotic-resistant bacteria [15]. Chlorophyllin is a water-soluble analog of the green pigment chlorophyll extracted from different plants such as spinach, green cabbage, grass, and dandelion [16]. Spinach-derived Chlorophyllin exhibited antimicrobial potential against bacteria such as *Bacillus subitilis* [15] and *Salmonella enterica* in combination with other antimicrobial agents [17]. *Mimosa pudica* derived Chlorophyllin showed antimicrobial potential against clinically significant drug-resistant pathogens, including *Escherichia coli*, *Klebsiella pneumonia*, *Staphylococcus aureus*, *Pseudomonas erogenous*, and *Proteus vulgaris* [18]. Chlorophyllin is also used to fabricate nanomaterials; Chlorophyllin-functionalized graphene oxide nanostructures show antimicrobial behavior against *E. coli* [19].

Considering Chlorophyllin’s antimicrobial potential, this study evaluated its antibacterial and antibiofilm activity against dental carries associated with *S. mutans*. Additionally, molecular docking studies were performed to investigate the mechanism of action of Chlorophyllin, and ADMET analysis was performed to assess its safety profile.

## 2. Materials and Methods

### 2.1. Microorganisms

Different dental swab samples of patients suffering from dental caries were collected at District Headquarters Hospital Kohat, Pakistan; placed in sterile tubes containing Brain Heart Infusion broth; and transported aseptically to the Department of Microbiology. The samples were cultured on Tryptone Yeast Extract Cysteine Sucrose Bacitracin (TYCSB) agar at 37 °C in a 5% CO_2_ incubator (ESCO, Singapore). Gram staining and biochemical tests were performed to identify and confirm *S. mutans* isolates.

### 2.2. Extraction and Purification of Chlorophyllin

Fresh leaves of *Spinacia oleracea* were collected locally and authenticated at the Department of Botany, Kohat University of Science and Technology, Kohat, Pakistan. Spinach leaves were washed properly, dried, and ground. Chlorophyll extraction was carried out with 96% methanol at 65 °C in a water bath. To prevent the conversion of chlorophyll into pheophytin, CaCO_3_ (0.1 M) was added. To remove leaf debris, the centrifugation of the extract was carried out. Following centrifugation, extract was filtered through Whatman filter paper. To avoid the photodegradation of chlorophyll due to oxidative stress, all the procedures were conducted under dark conditions. Chlorophyll filtrate was transferred into a separating funnel by mixing well with petroleum benzene. The separating funnel was placed undisturbed to allow for the separation of two phases. The chlorophyll migrated into the upper lipophilic benzene phase, which was treated with methanolic KOH (1 M). This resulted in the conversion of chlorophyll into water-soluble Chlorophyllin. Chlorophyllin, was separated from the benzene phase and shifted into another funnel. Excessive solvents were removed from Chlorophyllin using a rotary evaporator to obtain concentrated extract placed in dark conditions at 4 °C [20].

### 2.3. Antibacterial Potential of Chlorophyllin against S. mutans

In vitro, the antimicrobial activity of Chlorophyllin against *S. mutans* was determined using an agar well diffusion assay. Each sample was inoculated in 10 mL nutrient broth media and incubated for 24 h. After overnight incubation, *S. mutans* inoculum was spread onto Muller–Hinton agar plates using a sterile cotton swab. Next, 100 µL volume of Chlorophyllin extract was propelled into the wells, and the plates were incubated overnight at 37 °C to assess its antibacterial potential. Antibiotic Clindamycin was used as a positive control, and sterile distilled water was used as a negative control [21].

### 2.4. Biofilm Screening and Its Inhibition by Chlorophyllin

All ten *S. mutans* isolates were screened for their biofilm-forming properties using a test tube method. Each bacterial isolate was inoculated in 2 mL Brain Heart Infusion broth supplemented with 1% glucose. Following incubation at 37 °C for 24 h, test tubes were inverted, rinsed well with Phosphate Buffer Saline, and air-dried. Next, the test tubes were stained with 0.1% crystal violet for ten minutes and washed with sterilized distilled water to remove excess coloration. Chlorophyllin was added with bacterial culture to each test tube to determine the antibiofilm activity, and it was covered with aluminum foil to prevent photodegradation. After adding 1 mL of 95% ethanol in the stained test tubes for 10–15 min, biofilm strength was recorded based on their optical density at a 600 nm wavelength. Activity was performed in triplicate, the data were averaged, and the standard deviation was estimated.

### 2.5. Characterization of EPS by FTIR and Its Inhibition by Chlorophyllin

A 20 µL inoculum of *S. mutans* was added to 50 mL of sterile nutrient broth and incubated at 37 °C in 5% CO_2_ for 24 h at 140 rpm in a shaking incubator. Next day, the centrifugation of samples was carried out at 12,000 rpm for 20 min at 4 °C. Supernatants were collected, precipitated by adding 3 volumes of chilled ethanol, and kept overnight at 4 °C. The following day, the incubated sample was re-centrifuged at 5000 rpm for 30 min, and the pellets were collected and air-dried at room temperature. To assess the inhibition of EPS, 100 µL of Chlorophyllin was added to 50 mL of nutrient broth with a 20 µL culture of *S. mutans* while repeating the same methodology as used above. The EPS was characterized by using a Fourier Transform Infrared Spectrometer (Thermo Fisher Scientific, Waltham, MA, USA). The spectra of selected EPS samples were recorded using model Spectrum Two with the serial number 103385, i.e., Detector LITa03 (Laser Components, Olching, Germany). Potassium bromide was mixed with EPS samples to dilute and homogenize. The spectra were determined at a range of 500–3500 cm^−1^.

### 2.6. Molecular Docking Studies and Pharmacokinetics Properties of Chlorophyllin

**Figure 1:**
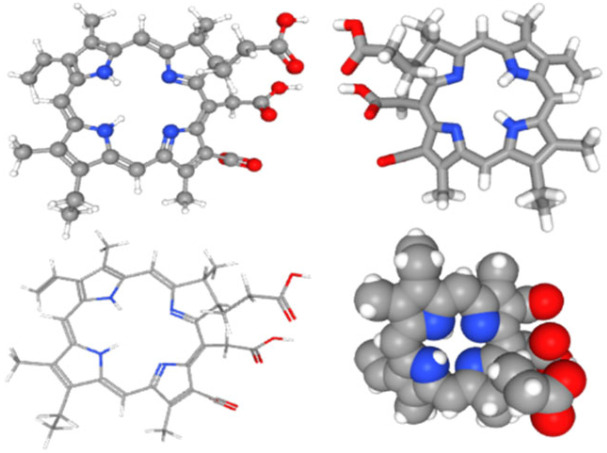
Schematic images of various Chlorophyllin structures were obtained from PubChem (https://pubchem.ncbi.nlm.nih.gov, accessed on 5 May 2024) [24].

**Figure 2:**
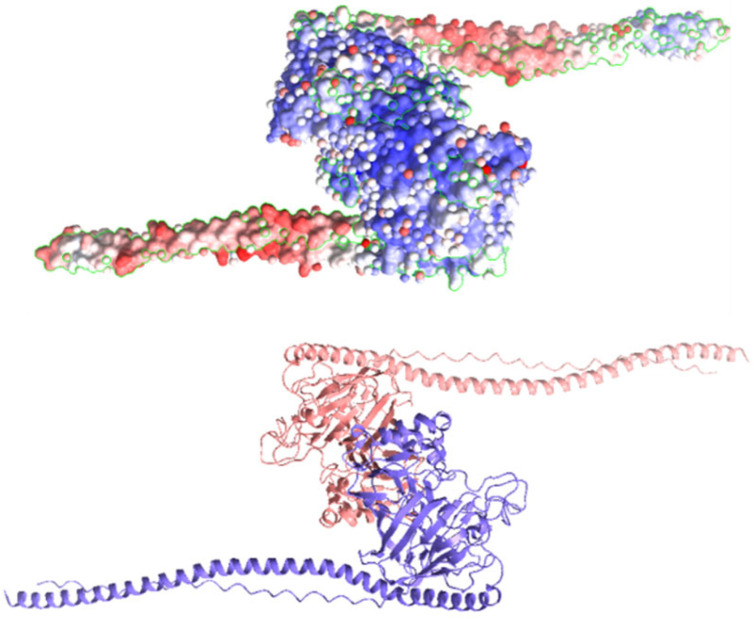
Schematic images of various AgI/II protein structures were obtained from (https://www.rcsb.org/, accessed on 5 May 2024) PDB ID: 3IPK [25].

Chlorophyllin was docked with the AgI/II protein of *S. mutans*, facilitating its adhesion and biofilm formation. The chemical structure and canonical SMILES format of Chlorophyllin (CID: 123798) were obtained from PubChem (https://pubchem.ncbi.nlm.nih.gov, accessed on 5 May 2024) (Figure 1), and three-dimensional crystal structures (PDB ID: 3IPK) of Ag I/II) were obtained from (https://www.rcsb.org/, accessed on 5 May 2024) (Figure 2). Structure preparation, binding pocket selection, and molecular docking were carried out using MOE version 2019.0102 (Molecular Operating Environment) [22]. MOE finds all possible binding geometries of receptor–ligand interactions based on a numerical score known as S-score. Binding affinities or S-scores are based on hydrogen bonds, salt bridges, hydrophobic interactions, cation–π interactions, and solvent exposure [23].

### 2.7. ADMET Analysis

ADMET analysis of Chlorophyllin was performed using ADMETlab 2.0 (https://admetmesh.scbdd.com/, accessed on 5 May 2024). Regarding ADMET, approximately 40% of drug candidates fail to pass clinical trials during the in silico study of adsorption, distribution, metabolism, excretion, and toxicity (ADMET) analyses [26].

## 3. Results

**Figure 3:**
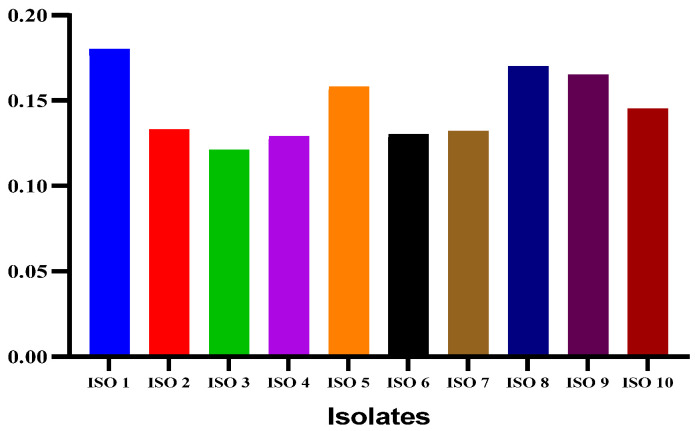
Variations in biofilm production by isolates of *S. mutans*.

A total of 10 *S. mutans* isolates were identified based on Gram staining and confirmed through culture and biochemical characteristics. The isolates were labeled as ISO 1, ISO 2, ISO 3, and ISO 10 (Figure 3). Biofilm screening revealed that all the isolates could form biofilms according to spectrophotometric results at 595 nm.

### 3.1. Chlorophyllin Potential against S. mutans

**Figure 4:**
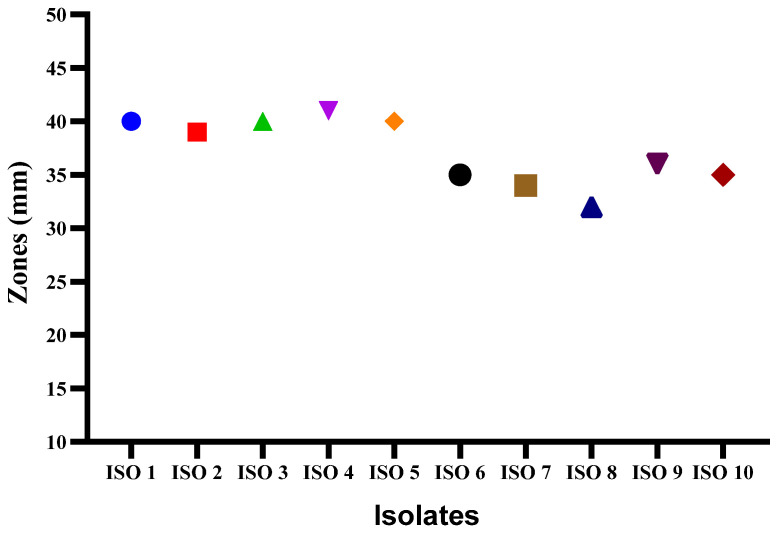
Illustrating zones of inhibition induced by Chlorophyllin extract against *S. mutans*.

An agar well diffusion assay revealed that all *S. mutans* isolates were susceptible to Chlorophyllin extract. The zones of inhibition measured indicated a significant antibacterial effect (Figure 4), with the most extensive zone reaching 40 ± 1.1 mm in diameter against ISO 4. Other notable inhibition zones had diameters of 40 mm against ISO 1, ISO 3, and ISO 5; 39 mm against ISO 2; 36 mm against ISO 9; 35 mm against ISO 6 and ISO 10; 34 mm against ISO 7; and 32 mm against ISO 8, respectively.

### 3.2. Antibiofilm Activity of Chlorophyllin

**Figure 5:**
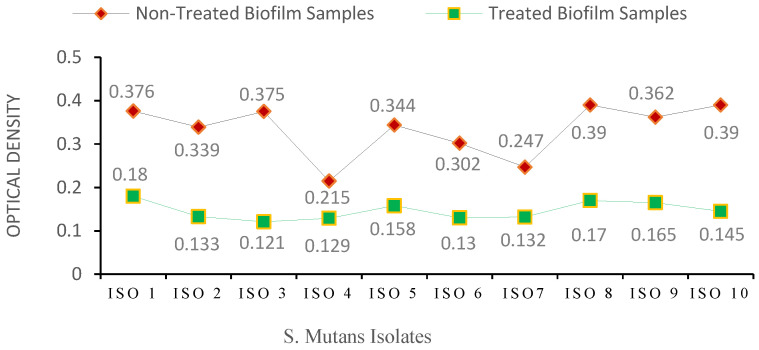
Comparative analysis of biofilm formation by *S. mutans* isolates before and after Chlorophyllin treatment based on *OD*_600_.

**Figure 6:**
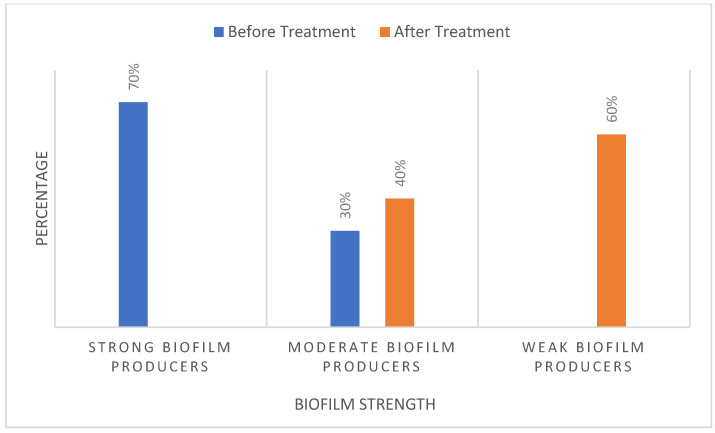
Relative percentages of *S. mutans* isolates producing robust, moderate, and weak biofilms before and after treatment with Chlorophyllin extract.

The *S. mutans* isolates showed different variations in biofilm formation (Table 1). The classification of biofilm formation and the corresponding number of bacterial isolates are presented in Table 2. Chlorophyllin showed a notable reduction in biofilm formation by *S. mutans*. Among the ten isolates, the strengths of four isolates of *S. mutans* (ISO 1, ISO 5, ISO 8, and ISO 9) were reduced from strong to moderate biofilms based on their optical densities of 0.18, 0.158, 0.170, and 0.165, respectively. In comparison, six isolates were reduced to weak biofilms with OD values of 0.133, 0.121, 0.129, 0.130, 0.130, 0.132, and 0.145 for ISO 2, ISO 3, ISO 4, ISO 6, ISO 7, ISO 7, and ISO 10, respectively, as shown in Figure 5/Table 3. The percentage strengths of biofilm-producing *S. mutans* samples before and after treatment with Chlorophyllin are mentioned in Figure 6.

### 3.3. Effect of Chlorophyllin on EPS Production Based on Strong Biofilm Formation

**Figure 7:**
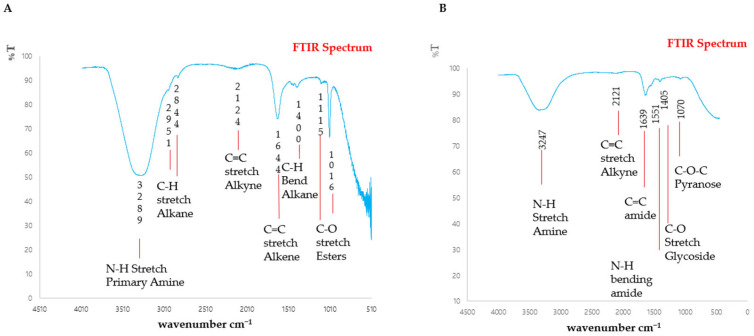
FTIR spectrum of EPS production by *S. mutans*: (**A**) non-treated sample, (**B**) Chlorophyllin-treated EPS Sample.

Treatment with Chlorophyllin reduced the number of corresponding functional groups. In Figure 7A, the FTIR spectrum of the non-treated sample showed a band intensity at 3289 cm^−1^, indicating N-H Stretch Primary Amine; the peak values at 2951 cm^−1^ and 2844 cm^−1^ show C-H stretch Alkanes, 2124 cm^−1^ shows a C≡C stretch Alkyne, and 1644 cm^−1^ shows that a C=C stretch Alkene is present. At 1400 cm^−1^, N-H Bend amides are revealed. C-O stretches of Esters are present at 1115 cm^−1^ and 1016 cm^−1^. In Figure 7B, the Chlorophyllin-treated sample shows an N-H Stretch Amine, a C≡C stretch Alkyne, a C=C Amide, an N-H bending Amine, a C-O Stretch Glycoside, and a C-O-C Pyranose at peak values of 3247 cm^−1^, 2121 cm^−1^, 1639 cm^−1^, 1551 cm^−1^, 1405 cm^−1^, and 1070 cm^−1^.

### 3.4. FTIR Analysis of EPS Production by S. mutans Based on Moderate Biofilm Formation

**Figure 8:**
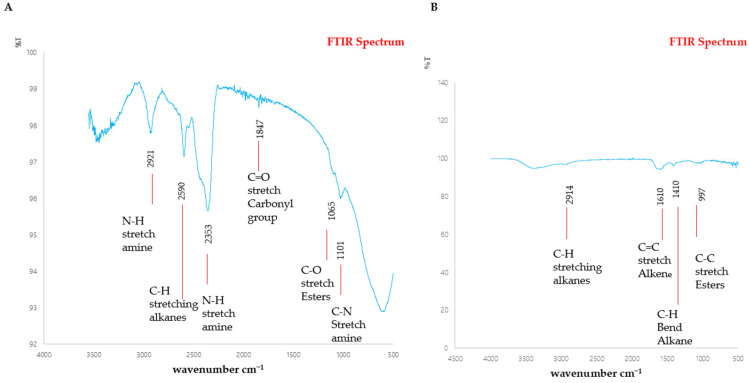
FTIR spectrum of EPS production by *S. mutans*: (**A**) non-treated sample; (**B**) Chlorophyllin-treated EPS sample.

The FTIR spectrum in Figure 8A shows a non-treated sample based on moderate biofilm. The peaks in the non-treated sample correspond to an N-H Stretch amine, a C-H Stretching alkane, an N-H stretch amine, a C=O stretch Carbonyl group, and a C-O stretch ester and a C-N stretch amine at band intensities of 2921 cm^−1^, 2590 cm^−1^, 2353 cm^−1^, 1847 cm^−1^, 1065 cm^−1^, and 1101 cm^−1^. In the CHL-treated sample, as shown in Figure 8B, C-H stretching Alkanes, a C=C stretch Alkene, a C-H bend Alkane and a C-C stretch Ester are revealed at different band intensities of 2914 cm^−1^, 1610 cm^−1^, 1410 cm^−1^, and 997 cm^−1^, respectively.

### 3.5. Molecular Docking Studies

**Figure 9:**
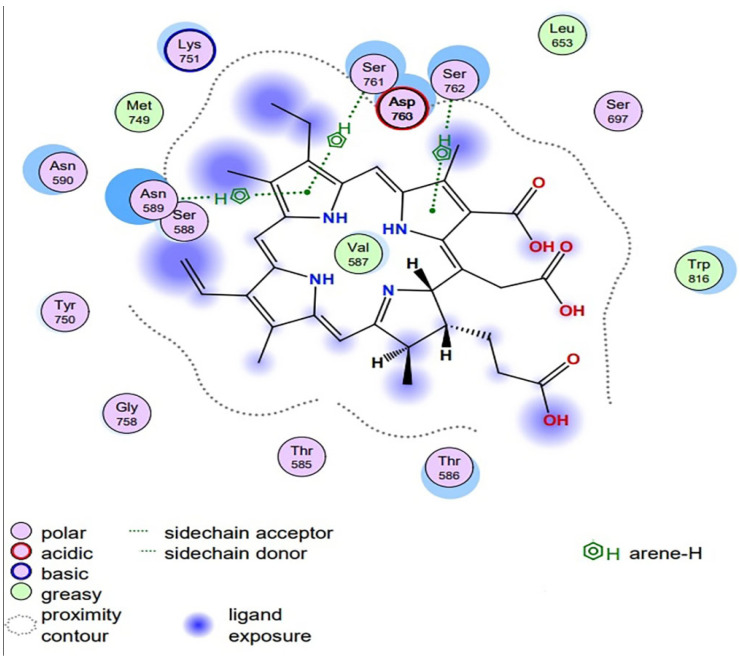
Two-dimensional interaction between Chlorophyllin and Ag I/II proteins was performed using the MOE and docking scores.

**Figure 10:**
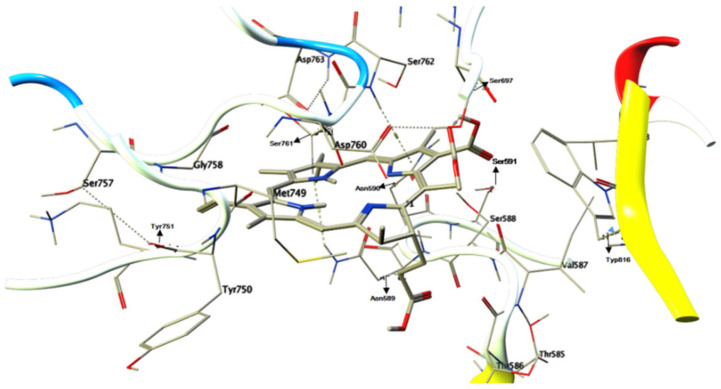
Three-dimensional interaction between Chlorophyllin and Ag I/II proteins.

Molecular docking results revealed that Chlorophyllin binds with *S. mutans* Ag I/II proteins, as shown in Figure 9 and Figure 10. The interaction between Chlorophyllin and Ag I/II proteins was performed using the Molecular Operating Environment (MOE). For docking London dG and GBVI/WSA dG (Generalized-Born Volume Integral/Weighted Surface area), scoring functions producing S-scores in units of kcal/mol were used in MOE, as shown in Figure 8. The Chlorophyllin-based negative Gibbs score of −9.8261 and RMSD (Root Mean Square Deviation) showed potential inhibitory activity of 1.7033. The Chlorophyllin “5-ring” interacted with ASN 589, SER 761, and SER 762 receptor residues through pi-H interactions, with 4.89 Ångstroms. “N 10” interacted with the receptor residue of SER 762 through a hydrogen bond (H-donor), with 2.99 Ångstroms, while “O 2” interacts with the receptor residue of ARG 824 through a hydrogen bond (H-acceptor), with 2.82 Ångstroms. Overall, the Chlorophyllin interaction’s S-score suggests that its inhibitory properties are supported by an RMSD of less than two, which predicts the alignment of the ligand pose within the binding pocket.

### 3.6. In Silico Pharmacokinetics Analysis of Chlorophyllin

ADMET analysis of Chlorophyllin performed via ADMETlab 2.0 revealed several important insights about physicochemical properties; Chlorophyllin exhibited a molecular weight of 618.23, with a density of 0.95 and a volume of 650.468. There were ten hydrogen bond acceptors with no donors, seven rotatable bonds, and five rings. Chlorophyllin demonstrated favorable drug-likeness with a QED value of 0.283, and the molecule’s Fsp3 value of 0.324 suggested good solubility. Chlorophyllin had optimal permeability with a Caco-2 score of −5.525 and was predicted to be a P-gp substrate and inhibitor. Overall, human intestinal absorption of Chlorophyllin was moderate. The distribution of Chlorophyllin within plasma protein binding (PPB) was 98.21%, with normal volume distribution (VD), along with low blood–brain barrier penetration. Chlorophyllin had minimal inhibitory activity with cytochrome P450, indicating a low likelihood of metabolic interactions. The excretion of Chlorophyllin exhibited average clearance and a short half-life. At the same time, the toxicity of Chlorophyllin was inactive as an hERG blocker and demonstrated positive human hepatotoxicity and drug-induced liver injury (DILI) probabilities. Chlorophyllin exhibited favorable pharmacokinetics properties, suggesting its potential suitability for further investigation as a therapeutic agent.

## 4. Discussion

Dental caries, a prevalent oral health issue, is primarily caused by the formation of biofilms by *S. mutans*, which are challenging to eradicate and contribute to their antimicrobial resistance. This research aimed to investigate the efficacy of Chlorophyllin against *S. mutans* and its biofilms, as well as its interaction with proteins of *S. mutans* through in silico studies, and assess the safety profile of Chlorophyllin using ADMET analysis.

In an agar well diffusion assay, Chlorophyllin extract (100 µL) exhibited varied inhibition zones against all tested isolates of *S. mutans*, indicating its antimicrobial solid potential. The recorded inhibitory zones in the agar well diffusion assay ranged from 32 mm to 40mm. Rajalakshmi et al. investigated the antibacterial activity of Chlorophyllin extracted from *Mimosa pudica* Linn against several clinically meaningful bacteria [18]. They recorded inhibitory zones of 9 mm to 18 mm at different concentrations of 25–100 μg/mL. Kang et al. (2013) reported that CHL had an inhibitory effect on the growth of *Propionibacterium acnes* with a MIC value of 100 μM [28]. Similarly, M and Banu et al. reported the antimicrobial activity of *Phyllanthus emblica*-extracted Chlorophyllin against *Staphylococcus aureus*, *Pseudomonas aeruginosa*, *Klebsiella pneumonia*, *Escherichia coli*, and *Candida albicans* [29]. Chlorophyllin showed promising inhibition of *Klebsiella pneumoniae* at 25–100 µL/well. The researchers also used CHL in coatings with other compounds and metals.

Zhang et al. (2022) reported the in vitro antibacterial activities of a Copper-bearing Chlorophyllin-induced Ca–P coating on the magnesium alloy AZ31 [30]. In their study, a Sodium Copper Chlorophyllin (SCC) with a porphyrin ring-induced Ca–P coating was prepared on AZ31 magnesium alloy, showed significant antimicrobial activity against *E. coli* and *S. aureus*, and was corrosion-resistant. The metallochlorophyllin (Tin Chlorophyllin) extracted from the leaves of *Morinda citrifolia* L. was used against several drug-resistant pathogens, namely *Salmonella typhi* and *Pseudomonas aeroginosa* [18]. Chlorophyllin can also be coated with polymers such as Chitosan for better antimicrobial efficacy [17]. The Chlorophyllin–Chitosan conjugate (Chl–CHS) exhibited antimicrobial effects against the foodborne pathogens *L. monocytogenes* and *E. coli* by the 7-log reduction in *L. monocytogenes* and 4.5-log reduction in *E. coli*. A comparative analysis showed that soaking strawberries in 200 ppm NaOCl reduced yeast/microfungus populations by 0.6 log. Additionally, the Chl-CHS coating protected strawberries from fungal contamination. Suresh et al. showed that Copper–Chlorophyllin and Sodium–Copper–Chlorophyllin synthesized from *Aloe vera* leaves have potent antimicrobial effects against *S aureus*, *B. cereus*, and *S typhi* [31].

Furthermore, we showed that Chlorophyllin is effective against the biofilms of *S. mutans* as it reduces 60% of the biofilms of *S. mutans* isolates. *S. mutans* produces EPS as a virulence factor [32], characterized by using an FTIR after treatment with Chlorophyllin. A change in FTIR spectra was observed as the number of functional groups was reduced, and new peaks of amide, glycoside, and pyranose were observed in the FTIR Spectrum of CHL-treated EPS samples. EPS is produced chiefly by bacterial Glucosyltransferases (GTFs), and its inhibition weakens the adhesion of bacteria to the surfaces [33]. Afrasiabi et al. demonstrated the potential effect of using a sub-lethal dose of antimicrobial photodynamic therapy sPDT with a diode laser plus a Chlorophyllin–Phycocyanin mixture (PhotoActive^+^) on changes in the *gtfB* gene in *S. mutans* [34]. In their study, sPDT using 2.4 × 10^−3^ mol/L PhotoActive^+^ with a 3 min irradiation time diode laser with an energy density of 104 J/cm^2^ significantly reduced *gtfB* gene expression at a rate of 3.5-fold (*p* < 0.05). Also, PhotoActive^+^-mediated sPDT demonstrated a significant reduction in the GtfB protein expression of *S. mutans* of up to 54% (*p* < 0.05). Afrasiabi et al. (2020) investigated the antibiofilm and antimetabolic effects of a Chlorophyllin–Phycocyanin (CHL-PC) mixture against *Streptococcus mutans* [34]. Their results indicated that at a maximum concentration of CHL-PC (5000 μg/mL) and a DL irradiation time of 3 min (103.12 J/cm^2^), the ex vivo cariogenic biofilm of *S. mutans* was reduced by 36.93% (*p* < 0.05). Their findings suggest that the CHL-PC mixture is safe for use in the oral environment. Similarly, Chlorophyllin-based antimicrobial photodynamic therapy showed inhibitory effects against the *Acinetobacter baumannii* biofilms [17].

Furthermore, we showed that Chlorophyllin potentially binds with the adhesins of *S. mutans*, which play an essential role in biofilm formation and dental caries [9]. The Chlorophyllin-based negative Gibbs score of −9.8261 and RMSD of 1.7033 showed potential inhibitory activity. The Chlorophyllin “5-ring” interacts with ASN 589, SER 761, and SER 762 receptor residues through pi-H interactions, with 4.89 Ångstroms. “N 10” interacts with the receptor residue of SER 762 through a hydrogen bond (H-donor), with 2.99 Ångstroms, and “O 2” interacts with the receptor residue of ARG 824 through a hydrogen bond (H-acceptor), with 2.82 Ångstroms. Overall, the S-scores of Chlorophyllin interactions suggest that their inhibitory properties are supported by an RMSD of less than two. According to our knowledge, we are the second researchers to use antigen I/II as a target for antimicrobials, as the first study was reported by Rivera-Quiroga et al., who showed Ag I/II targets adhesins for virtually screened inhibitory molecules in in silico studies [9].

Additionally, ADMET analysis of Chlorophyllin showed safety, as demonstrated by favorable drug-likeness, good solubility, moderate human intestinal absorption, and low blood–brain barrier penetration. Chlorophyllin had minimal inhibitory activity with cytochrome P450, and the toxicity of Chlorophyllin was inactive as an hERG blocker, which demonstrated positive human hepatotoxicity and DILI probabilities. Moreover, Chlorophyllin is easily available, relatively inexpensive, and straightforward compound. These qualities make Chlorophyllin an attractive option for various applications including biomedical coatings [35,36].

## 5. Conclusions

This study has demonstrated the significant antibacterial effects of Chlorophyllin against *S. mutans* and its biofilms. The in vitro experiments revealed a reduction in biofilm formation, highlighting the potential of Chlorophyllin as an effective agent in combating dental caries. In silico studies further supported these findings by showing binding solid affinity between Chlorophyllin and the adhesin proteins of *S. mutans*, suggesting a possible mechanism for its antibiofilm activity. Additionally, ADMET analysis confirmed the safety profile of Chlorophyllin, reinforcing its suitability for oral health applications. These results underscore the promise of Chlorophyllin as a safe and potent antimicrobial agent against *S. mutans*, paving the way for its potential use in therapeutic formulations to prevent and control dental biofilms and caries.

## Figures and Tables

**Table 1:** The biofilm-forming ability of *S. mutans* isolates.

*S. mutans* Isolates	Biofilm Strength	Mean ± Standard Deviation
ISO 1	Strong Positive	0.376 ± 0.11
ISO 2	Strong Positive	0.339 ± 0.10
ISO 3	Strong positive	0.375 ± 0.11
ISO 4	Moderate positive	0.215 ± 0.06
ISO 5	Strong positive	0.344 ± 0.14
ISO 6	Moderate positive	0.302 ± 0.06
ISO 7	Moderate positive	0.247 ± 0.05
ISO 8	Strong positive	0.390 ± 0.16
ISO 9	Strong positive	0.362 ± 0.11
ISO 10	Strong positive	0.390 ± 0.16

**Table 2:** Biofilm formation classification based on optical density (OD) and distribution of bacterial isolates [27].

Biofilm Classification	Calculated OD Range	Number of Bacterial Isolates
Non-Adherent	<0.077	-
Weak	0.077–0.154	-
Moderate	0.154–0.308	3 (30%)
Strong	>0.308	7 (70%)

**Table 3:** The biofilms’ strengths after Chlorophyllin treatment.

*S. mutans* Isolates	Biofilm Strength	Mean ± Standard Deviation
ISO 1	Moderate positive	0.18 ± 0.04
ISO 2	Weak positive	0.133 ± 0.01
ISO 3	Weak positive	0.121 ± 0.16
ISO 4	Weak positive	0.129 ± 0.02
ISO 5	Moderate positive	0.158 ± 0.02
ISO 6	Weak positive	0.130 ± 0.02
ISO 7	Weak positive	0.132 ± 0.04
ISO 8	Moderate positive	0.170 ± 0.17
ISO 9	Moderate positive	0.165 ± 0.05
ISO 10	Weak positive	0.145 ± 0.02

## Data Availability

The original contributions presented in this study are included in this article; further inquiries can be directed to the corresponding author.
